# High spatial resolution scintillator dosimetry of synchrotron microbeams

**DOI:** 10.1038/s41598-019-43349-6

**Published:** 2019-05-03

**Authors:** James Archer, Enbang Li, Jeremy Davis, Matthew Cameron, Anatoly Rosenfeld, Michael Lerch

**Affiliations:** 1 Centre for Medical Radiation Physics, University of Wollongong, Wollongong, NSW 2522 Australia; 2 Illawarra Health and Medical Research Institute, University of Wollongong, Wollongong, NSW 2522 Australia

**Keywords:** Fibre optics and optical communications, Radiotherapy

## Abstract

Microbeam radiation therapy is a novel pre-clinical external beam therapy that uses high-brilliance synchrotron X-rays to deliver the necessary high dose rates. The unique conditions of high dose rate and high spatial fractionation demand a new class of detector to experimentally measure important beam quality parameters. Here we demonstrate the highest spatial resolution plastic scintillator fibre-optic dosimeter found in the literature to date and tested it on the Imaging and Medical Beam-Line at the Australian Synchrotron in a X-ray beam where the irradiation dose rate was 4435 Gy/s. With a one-dimensional spatial resolution of 10 *μ*m the detector is able to resolve the individual microbeams (53.7 ± 0.4 *μ*m wide), and measure the peak-to-valley dose ratio to be 55 ± 17. We also investigate the role of radioluminescence in the optical fibre used to transport the scintillation photons, and conclude that it creates a significant contribution to the total light detected.

## Introduction

Synchrotron microbeam radiation therapy (MRT) is a novel external beam cancer therapy currently in the pre-clinical research stage. The highly collimated and high brilliance X-rays required for MRT can only be produced in a synchrotron. Despite the relatively small number of synchrotron facilities world-wide, there is emerging research into compact synchrotron technologies^1–3^ which can allow MRT to become a widespread treatment of currently untreatable cancers^4,5^. The current hypothesis for the high resistance to damage of the healthy tissue is that the vasculature is unaffected by microbeams ranging from 25 *μ*m to 100 *μ*m width and 200 *μ*m to 400 *μ*m spacing, while the tumour vasculature is destroyed^6–10^. Synchrotron x-rays are required for MRT due to their high collimation, to ensure the microbeam quality, and the high brilliance, to ensure the dose can be delivered quickly enough to minimise dose-blurring from patient movement. Independent detector technologies must be developed to provide essential quality assurance of the X-ray beam and treatment plan. An overview of devices capable of MRT dosimetry has been presented previously^11^, hence here we will only present a summary high spatial resolution real time dosimeters that are capable of MRT dosimetry.

Radiochromic film is considered a dosimetric standard, but is limited in its application to MRT. The high range of doses between the peaks and valleys means that two exposures of different duration on separate film are required to measure the peak and valley doses. Further, it has been shown that film measurements can be inaccurate to up to 15%^12^. Metal oxide semiconductor field-effect transistors (MOSFET) have the required spatial resolution for MRT dosimetry, but lack the radiation hardness to withstand the high dose rate^13^. A number of silicon strip detectors (SSD) have been developed for MRT beam quality monitoring^14–16^. These detectors have reached an estimated resolution of 15 *μ*m defined by the electrical field distribution under biasing.

A commercial single crystal diamond detector, the PTW microDiamond, as also been applied to MRT fields due to its very high spatial resolution. It claims a 1 *μ*m one-dimensional sensitive volume (with 2.2 mm diameter in the other dimensions)^17^.

Scintillator dosimeters have been applied to a variety of contexts. LINAC photon dosimetry has been done with a variety of detectors, including plastic plastic scintillators (which are advantageous due to their water equivalence^18–20^) and inorganic dosimeters (which are advantageous due to their superior light output and radiation resistance, as well as characteristics that allow easy stem-effect filtering^21–24^). A preliminary study of Europium and Lithium doped yttrium oxide crystal scintillator has been applied to x-ray micro-fields, with limitations due to the non-water equivalence of the crystalline material^25^. In this work we will be focusing on plastic scintillator fibre-optic dosimeters (FODs). Plastic scintillator FODs are desirable due to their water equivalence, radiation hardness and energy independence^18,26^. Specifically, the radiation hardness will be an important factor for MRT dosimetry. Beddar *et al*. found that their plastic scintillator FOD saw a 2.8% decrease in sensitivity after exposure to 10 kGy of Cs-137 gamma rays^18^. This level of radiation hardness is appropriate for exposures to MRT x-rays with minimal detector sensitivity deviations. While an inorganic solution (such as a crystal detector^21^ or doped-silica detector^24^) would provide a smaller sensitivity to radiation damage, the primary focus of this work is to develop a dosimeter that is as water equivalent as possible for MRT. Typically, scintillator dosimeters are applied when high spatial resolution is not required, because plastic scintillators have a low light yield (typically 11,000 photons/MeV^27^) and so the dosimeter sensitivity is limited by the small scintillator volumes required for high resolution dosimetry. However, the high brilliance of a synchrotron source provides a measurable response in scintillator dosimeters. Optical detectors have been applied to imaging microbeam X-rays in the past^25,28^, however these detectors have not been demonstrated and tested at highly brilliant synchrotron light source facilities.

One of the challenges with optical dosimetry is the so-called stem effect: light generated in the optical fibre that is not related to the dose deposited in the scintillator sensitive volume^18^. There are two sources of this stem light: Cherenkov radiation and radioluminescence. Due to the refractive index of the optical fibre core, there is no Cherenkov radiation generated under synchrotron X-ray irradiation^26^, instead only radioluminescence is present. It dominates at lower wavelengths^29^, and occurs less in PMMA-core optical fibres than silica-core optical fibres^30^.

We have previously tested FODs with MRT beams that have a spatial resolution of 50 *μ*m^31^ and 20 *μ*m^11^. In this work we demonstrate a FOD with a spatial resolution of 10 *μ*m, the highest of a plastic scintillator detector found in the literature.

## Materials and Methods

**Figure 1:**
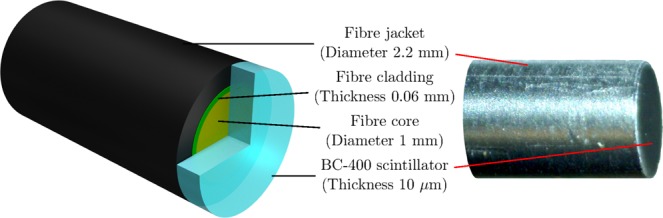
(Left) A cartoon of the scintillator fibre-optic dosimeter (not to scale). (Right) A microscope image of the tip of the probe.

The design of the scintillator fibre-optic dosimeter is summarised in Fig. 1. A 10 ± 2 *μ*m thick film of BC-400 plastic scintillator is optically coupled to a 1 mm diameter core Eska CK-40 optical fibre. The scintillator thickness defines the one-dimensional spatial resolution of the FOD probe as 10 *μ*m in the axial direction, and 1 mm in the radial direction (determined by the optical fibre core diameter). The scintillation light (peaking at 423 nm) is transported via optical fibre to a SensL MiniSM Silicon Photomultiplier 10035 (SiPM)^32^. The scintillator volume is 0.0380 mm^3^, while the collection volume (encompassed by the optical fibre acceptance cone) is 0.00785 mm^3^ Table 1.

**Table 1:** Some properties of BC-400 plastic scintillator and the comparison to water (At STP).

Quantity	BC-400	Water
Density (g/cm^3^)	1.023	0.998
Electron density (e/cm^3^)	3.37 × 10^23^	3.33 × 10^23^
Composition	Polyvinyltoluene [CH_2_CH(C_6_H_4_CH_3_)]_*n*_	H_2_O

Scintillator data from Saint-Gobain Crystals^27^.

The scintillation and SiPM collection efficiency spectra can be found in Archer *et al*.^31^. As the scintillator is not covered, experiments must be done in darkness to minimise the amount of ambient light entering the optical fibre. Measuring the background signal allows a simple subtraction to be done, but excess light will limit the dynamic range of the detector.

**Figure 2:**
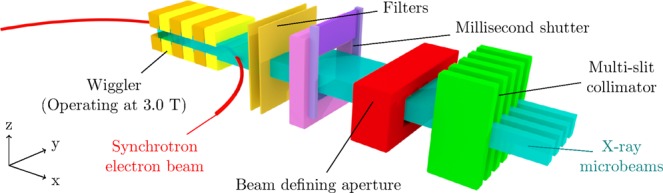
A cartoon of the IMBL at the Australian Synchrotron. Reproduced from Archer *et al*.^11^. Reproduced with permission of the International Union of Crystallography (https://journals.iucr.org/).

**Figure 3:**
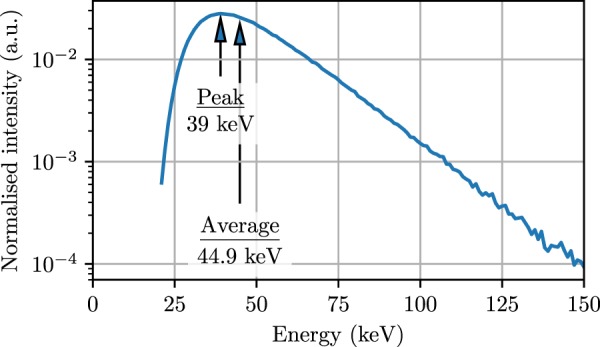
GEANT4 simulation of the IMBL to give the X-ray spectrum before entering the target region.

The measurements were performed on the Imaging and Medical Beam-Line (IMBL) at the Australian Synchrotron. The 3.032 GeV, 200.2 mA electron beam is subject to a 3.0 T wiggler magnetic field to produce synchrotron X-ray radiation (Fig. 2). These X-rays are filtered to control the spectrum and intensity, with filter combination F4 (as defined in Table 3, Stevenson *et al*.^33^). The spectrum is shown in Fig. 3. The beam was shaped with a beam defining aperture (BDA) to a width of 30 mm and a height of 2.014 mm, 1.052 mm or 0.532 mm, then was further shaped with a conformal mask to 20 mm × 20 mm. A tungsten multi-slit collimator (MSC) with gaps of 50 *μ*m and pitch 400 *μ*m is used to spatially fractionate the beam into microbeams. The dose rates of the various fields without the MSC in place are presented in Table 2.

**Table 2:** The intrinsic broadbeam dose rates measured with a PTW Pinpoint N31014 ionisation chamber, with the 20 mm × 20 mm conformal mask in place.

Field height (mm)	2.014	1.052	0.532
Dose rate (Gy/s)	4435	4441	4255

The FOD was mounted in edge-on mode (fibre axis perpendicular to the beam direction and parallel to the direction of fractionation) in a specially constructed PMMA holder, allowing the microbeam fractionation to be measured with a spatial resolution of 10 *μ*m, the thickness of the scintillator. By scanning the probe continuously across the beam at 1 mm/s, and sampling the SiPM response with an analogue front end at a high frequency (2000 Hz) a profile of the microbeams can be measured. The PMMA holder minimised alignment issues experienced with thin detectors such as this; the cross section exposed to the beam has thickness *t* given by:

1 $$t={t}_{0}\,\cos \,\theta +d\,\sin \,\theta \approx {t}_{0}+d\theta $$

where *t*_0_ is the one-dimensional spatial resolution (10 *μ*m), *d* is the diameter of the optical fibre core (1000 *μ*m) and *θ* is the misalignment angle in radians. For small angles, the increase in effective thickness is proportional to the core diameter, and hence is sensitive to misalignment. The PMMA holder will ensure that the detector is aligned within 1° in both rotational axis (about the *x* and *z* axes defined in Fig. 2). The *z* axis alignment can be further refined using the DynMRT rotation stage (by minimising the measured microbeam width), but there is currently no procedure possible with the current setup to align about the *x* axis.

## Results and Discussion

### Microbeam scans

**Figure 4:**
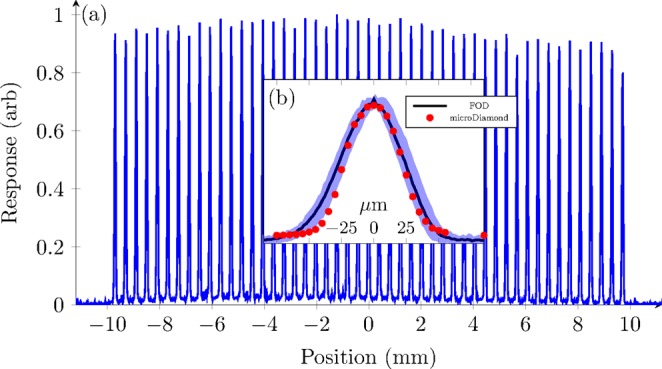
(**a**) 10 *μ*m probe microbeam scan. (**b**) The average of all the (normalised) microbeams, with the 95% confidence interval at each point. Also shown is the microDiamond measurement of a single microbeam (red).

**Figure 5:**
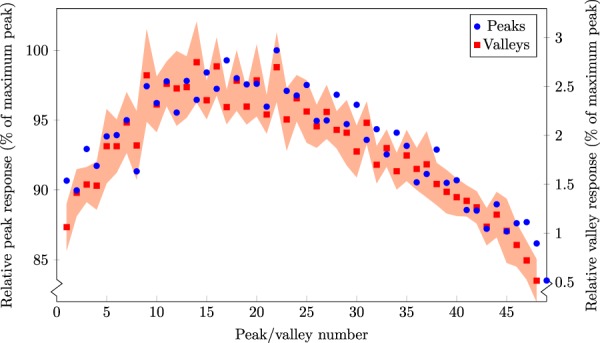
Peaks (left axis) and valleys (right axis) across the intrinsic microbeam array shown in Fig. 4. The standard deviation of the valley dose in each valley is presented as the red (shaded) region.

**Figure 6:**
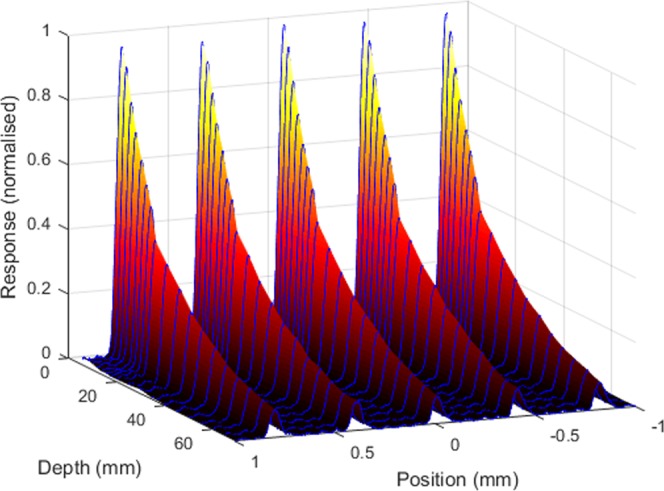
Microbeam depth dose from 6 mm to 70 mm depth (For full 3D view see Supplementary Fig. S1).

Figure 4 shows the intrinsic microbeam array scan (with 10-point moving average smoothing applied – this moving average covered a 5 *μ*m width). The inset shows the average and 95% confidence interval of all the (un-smoothed) microbeams. Figure 5 also shows the peak and valley values across the microbeam array. The peak height is very accurate and so no uncertainties are presented, while the valley values were averaged over a 200 *μ*m region between the peaks, and the standard deviation is shown. The full-width at half-maximum (FWHM) was calculated to be 53.7 ± 0.4 *μ*m. The peak-to-valley dose ratio (PVDR) over the entire microbeam array is 55 ± 17. Over the central 15 microbeams, the PVDR is 18.2 ± 1.5. This scan was repeated from 6 mm depth in water to 70 mm depth, which can be seen in Fig. 6. The step size is 2 mm up to 20 mm depth, and 5 mm for all deeper depths. For comparison, a microbeam scan was also done with a PTW microDiamond dosimeter, which has a spatial resolution of 1 *μ*m^17^. The PMMA holders used for both detectors were identical, except for the opening to hold the detectors due to their differing geometry.

The width of the microbeams is expected to be 50 *μ*m, which agrees well with the measured average FWHM of 53.7 ± 0.4 *μ*m. While this is greater than the intrinsic microbeam width, it is expected due to the dose blurring over the 10 *μ*m sensitive volume of the scintillator. This also validates the alignment of the detector to the incident X-rays. Measurements with the microDiamond detector gave a FWHM of 52.5 ± 5 *μ*m (which can be seen in Fig. 4). This agrees within uncertainty with the FOD. The large uncertainty here is due to the detector being stepped by 5 *μ*m between measurements. Interpolation between dose measurements allowed a more refined FWHM to be acquired. The microDiamond profile matches well with the FOD profile, with the exception of between −50 and −25 *μ*m positions, where the FOD response is slightly higher than the microDiamond. We believe this is due to the higher spatial resolution of the microDiamond. The agreement between the shape of both profiles suggests that the FOD is measuring an accurate beam profile with minimal dose-blurring. Alternatively, it is possible that the microDiamond effective sensitive volume is larger than the theoretical case, and so both detectors may be experiencing similar levels of dose-blurring. This may be due to their being an effect from the packaging around the diamond sensitive volume to keV x-rays. The microDiamond has a 2.2 mm diameter of sensitive volume, and so is more sensitive to misalignment then the FOD.

To evaluate the PVDR, the central microbeams are considered for consistency. There is a roll-off of dose in the valleys towards the edge of the microbeam array, giving a large difference in PVDR across the profile. This can be seen in both the peaks and valleys in Fig. 5. The asymmetry in this figure indicates a slight rotational misalignment in the MSC. The average PVDR of all microbeams at 6 mm depth is 55 ± 17. However, over the central 15 microbeams, where the valleys are much more consistent, the PVDR is 18.2 ± 1.5. This agrees well with the central microbeam being measured with the microDiamond giving 17.1 ± 0.8.

### Depth dose scans

**Figure 7:**
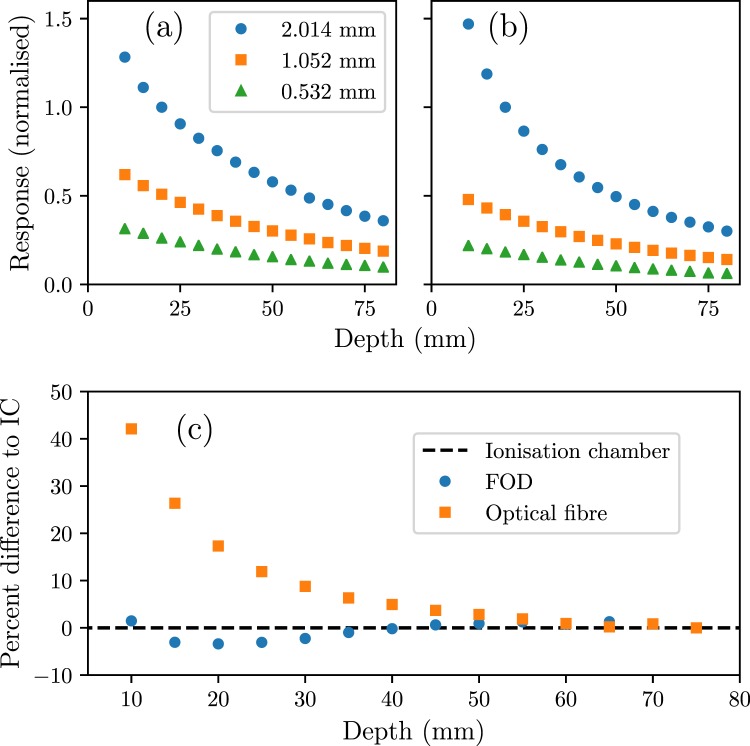
Broadbeam depth dose measured with three field heights defined by the BDA (legend), normalised to 20 mm depth. (**a**) Shows the results for the FOD, while (**b**) shows the response from just the optical fibre. (**c**) Shows the relative difference of both responses defined by the 2.014 mm BDA to a PTW Pinpoint N31014 ionisation chamber.

With the FOD mounted in edge-on mode, the probe was scanned vertically in the *z* direction (defined in Fig. 2) through the HDR broadbeam (with no MSC fractionating the field) to measure how the broadbeam dose changes with depth (depth dose). This was repeated at smaller field heights of 1.052 mm and 0.532 mm. This methodology is consistent with other dosimetry devices (see Fournier *et al*. 3.1^34^). The SiPM charge was measured with a commercial electrometer, PTW UNIDOSwebline, used for dosimetry. The charge was integrated over the duration of the scan, allowing relative dose to be measured. The dark current was too high to allow the electrometer to zero the readings (due to signal saturation on the highest sensitivity) so a “dark scan” with no X-rays allowed this to be characterised and subtracted in analysis. Figure 7 shows the depth dose measured with the FOD, along with the response from just the optical fibre. The response in the optical fibre is due to radioluminescence in the fibre.

It can be seen in Fig. 7(b) that the response with only optical fibre in the field has a higher relative response at low depths than the scintillator and fibre together, Fig. 7(a). This over-response is around 40% higher than the FOD signal at the same depth. Further, this effect is more significant with larger field heights. It is not due to high dose rate due to the effect also being seen at much lower dose rates^11,31^. The reduced discrepancy between the FOD signal and ionisation chamber, compared to the fibre-only signal, suggests that the discrepancy is minimal in the scintillator itself. As the optical fibre core is PMMA, the relative generation of radioluminescence is low, compared to silica core optical fibres^30^. However, it is still a significant part of the total light signal collected. We estimate this to be around 52% of the signal with the scintillator in the centre of the field, however this value is dependent on the exact fraction of fibre inside the field for this measurement. To adequately deal with this effect in future measurements, and any quality assurance methods, a secondary probe in parallel with the FOD measuring only the radioluminescence in the optical fibre can be used^18,19,26^. Other methods such as filtration or spectral separation are not efficient due to the strong overlap between the radioluminescence and scintillation spectra.

One of the primary challenges with using this dosimeter is the low light signal measured at the SiPM, which limits the applicability to high dose rates. We estimate that, using the ionisation chamber results at 75 mm depth and the low FOD response at this depth, that the minimum dose rate that can be confidently measured is 200 Gy/s. One method for increasing the light signal is to use an inorganic scintillator, which typically have a much higher yield. However, this will increase the dose perturbations due to the higher atomic mass elements used. Increasing the optical fibre core diameter will also increase the collected light, but will make the detector more sensitive to misalignment.

## Conclusion

In this work we have presented the highest spatial resolution plastic scintillator fibre-optic dosimeter found in the literature with a collection volume of 0.00785 mm^3^ and a one-dimensional spatial resolution of 10 *μ*m. Synchrotron X-ray microbeams have been resolved with this detector and measured the microbeam FWHM to be 53.7 ± 0.4 *μ*m. The detector is limited by radioluminescence in the optical fibre, with solutions to this being investigated. This detector has the potential to be applied to characterising highly brilliant synchrotron X-rays and quality assurance in microbeam radiation therapy.

## Supplementary information

S1
